# Usability and effectiveness of inhaled methoxyflurane for prehospital analgesia - a prospective, observational study

**DOI:** 10.1186/s12873-021-00565-6

**Published:** 2022-01-15

**Authors:** Helmut Trimmel, Alexander Egger, Reinhard Doppler, Mathias Pimiskern, Wolfgang G. Voelckel

**Affiliations:** 1 Department of Anaesthesiology, Emergency and Intensive Care Medicine, General Hospital, Corvinusring 3-5, A-2700 Wiener Neustadt, Austria; 2 Karl Landsteiner Institute for Emergency Medicine, Corvinusring 3-5, A-2700 Wiener Neustadt, Austria; 3 Christophorus Air Rescue, OeAMTC, Baumgasse 129, A-1030 Vienna, Austria; 4 Department of Anaesthesiology and Intensive Care Medicine, General Hospital Scheibbs, Eisenwurzenstraße 26, A-3270, Scheibbs, Austria; 5 Department of Anaesthesiology and Intensive Care Medicine, General Hospital Rottenmann, St. Georgen 2-4, A-8786, Rottenmann, Austria; 6 Department of Anaesthesiology and Critical Care Medicine, AUVA Trauma Centre Salzburg, Salzburg, Austria; 7 Paracelsus Private Medical University of Salzburg, Salzburg, Austria; 8 Department of Health Studies, University of Stavanger, Stavanger, Norway

**Keywords:** Inhaled analgesia, Emergency medical service, Methoxyflurane, Prehospital pain management, Paramedics

## Abstract

**Background:**

Pain relief in the prehospital setting is often insufficient, as the administration of potent intravenous analgesic drugs is mostly reserved to physicians. In Australia, inhaled methoxyflurane has been in routine use by paramedics for decades, but experience in Central European countries is lacking. Thus, we aimed to assess whether user friendliness and effectiveness of inhaled methoxyflurane as sole analgesic match the specific capabilities of local ground and air-based EMS systems in Austria.

**Methods:**

Observational study in adult trauma patients (e.g. dislocations, fracture or low back pain following minor trauma) with moderate to severe pain (numeric rating scale [NRS] ≥4). Included patients received a Penthrop® inhaler containing 3 mL of methoxyflurane (maximum use 30 min). When pain relief was considered insufficient (NRS reduction < 3 after 10 min), intravenous analgesics were administered by an emergency physician. The primary endpoint was effectiveness of methoxyflurane as sole analgesic for transport of patients. Secondary endpoints were user friendliness (EMS personell), time to pain relief, vital parameters, side effects, and satisfaction of patients.

**Results:**

Median numeric pain rating was 8.0 (7.0–8.0) in 109 patients. Sufficient analgesia (reduction of NRS ≥3) was achieved by inhaled methoxyflurane alone in 67 patients (61%). The analgesic effect was progressively better with increasing age. Side effects were frequent (*n* = 58, 53%) but mild. User satisfaction was scored as very good when pain relief was sufficient, but fair in patients without benefit. Technical problems were observed in 16 cases (14.7%), mainly related to filling of the inhaler. In every fifth use, the fruity smell of methoxyflurane was experienced as unpleasant. No negative effects on vital signs were observed.

**Conclusion:**

In prehospital use, inhaled methoxyflurane as sole analgesic is effective for transport of trauma patients (62%) with moderate to severe pain. Older patients benefit especially from inhaled methoxyflurane. Side effects are mild and vital parameters unaffected. Thus, inhaled methoxyflurane could be a valuable device for non-physician EMS personnel rescue services also in the central Europe region.

## Background

The treatment of acute pain is a central task in emergency medicine [1, 2]. However, the quality and quantity of pain management in practice are often criticised [3, 4]. Although in principle a range of various analgesic drugs are available, it seems that they are not deployed to their full potential: Insufficient training, insufficient experience in assessing pain intensity and applying analgesics [3, 4], and a lack of therapeutic standards are often mentioned as factors inhibiting their use [5]. Use of opioids may be limited due to concerns about relevant side effects and potential drug abuse, and in some jurisdictions (including Austria) by laws that reserve their use to emergency physicians. Non-opioid analgesics, on the other hand, are often associated with slower action and lower potency, though some studies suggest this may be mistaken [6–8]. Issues of insufficient training and experience may of course reflect that some drugs require specific skills in order to be handled successfully.

In other words, there is an unmet demand for analgesics that are easy and safe to use, ideally by paramedics, independent of emergency physicians [9]. Inhaled analgesics have considerable advantages in prehospital emergency medicine. They do not require an intravenous line, and the patients themselves can titrate the dosage: depending on the pain level, they can interrupt or increase the supply of the drug. The available substances – nitrous oxide and methoxyflurane – are fast acting, have hardly any critical side effects in experienced hands [10, 11] and may be as effective as opioids [12]. In English-speaking countries, nitrous oxide (Entonox®) is well-proven in decades of use both in the prehospital and hospital settings, in the latter in combination with opioids [10, 13–16].

Methoxyflurane, a volatile anaesthetic from the group of the di-alkyl esters (2-dichloro-1:1-difluoroethyl-methyl-ester) has been used at low, purely analgesic concentrations by paramedics in Australia (Penthrox®) since 1975, in New Zealand since 2002 and in several eastern European countries since 2010 [17–22]. By now, inhaled methoxyflurane has been studied in more than 200,000 adults and paediatric patients in registry data and prospective trials [19, 23, 24]. To date, there are no published reports of serious side effects, in particular malignant hyperthermia [25]. Nitrous oxide has barely been taken up in Central Europe (despite intensive efforts of the industry and multiple positive studies [26, 27]), while methoxyflurane was not even approved here until recently. The European Medicines Agency (EMA) authorization for methoxyflurane was only issued in 2018; in Austria it is approved as ‘Penthrop®’ (for therapy of moderate to severe pain. By now, besides the original trial for the authorization [28], methoxyflurane has been studied in further prospective, randomized trials from France, Italy and Spain, all carried out in in-hospital emergency departments [29–31]. However, it is not yet possible to make an assessment on prehospital use of methoxyflurane by paramedics in Europe [32].

The goal of our study was therefore to test the effectiveness of Penthrop® (inhaled methoxyflurane) as the sole analgesic in patients with moderate to severe pain following trauma. The study was performed by experienced emergency physicians. However, we wanted to explore whether Penthrop® could be an alternative in situations where an intravenous line cannot be established immediately, for example in alpine mountain rescue and whether methoxyflurane could be recommended for use by paramedics in the context of Central European prehospital emergency care.

## Methods

### Aims

The primary endpoint was effectiveness of Penthrop® as sole analgesic, measured as the proportion of cases in which sufficient analgesia was achieved by giving methoxyflurane alone. The following secondary endpoints were chosen: reduction of the initial pain level (arrival of the emergency physician = minute 0) based on the numeric rating scale (NRS) and the effects of the administered medication on the vital parameters circulation (pulse rate, blood pressure), oxygenation (SpO_2_) and consciousness level (GCS) each at 5, 10, 15, 20, and 30 min after beginning of treatment. The user friendliness of methoxyflurane in the prehospital setting (feasibility of the self-titration by the patient, handling of the drug by the rescue team) was scored on a 5-part Likert scale (1 = highly satisfactory, 2 = satisfactory, 3 = neutral, 4 = unsatisfactory, 5 = highly unsatisfactory). Additionally, the time until onset of the drug effect, the number of patients who required an intravenous line or supplemental analgesia, and the patient’s satisfaction with the analgesia and the care in general (5-part Likert scale, as above) were evaluated. Finally, any side effects of the medication were documented by proactively asking the patient, and, in addition, any technical problems were recorded.

### Design

Following approval by the Lower Austrian Ethics Committee (GS1-EK-4/577–2018), a prospective, non-randomized (non-interventional) Phase IV study was carried out.

### Setting

The drug was used by emergency physicians in accordance with its approval, mainly for fractures and/or dislocations of the upper or lower limbs at 6 ground-based and 6 air ambulance stations in Austria.

### Participants

Methoxyflurane (Penthrop®,) was administered as an alternative to intravenous analgesia to trauma patients *(*e.g. *fractures, dislocation or low back pain following minor trauma)* over 18 years old who had moderate to severe pain (NRS ≥4), were fully conscious and able to give informed consent, and had no impairment of vital functions. The contraindications and exclusion criteria listed in Table 1 were respected.

**Table 1:** In- and exclusion criteria for Methoxyflurane (Penthrop®) administration

Inclusion criteria	Exclusion criteria
• Age ≥ 18 years • Moderate-to-severe pain (NRS ≥ 4) secondary to minor trauma (e.g. fractures, luxation of big joints) • Conscious patients • Ability to give verbal informed consent	• Refusal of participation in this trial • Known personal or familial hypersensitivity to fluorinated anaesthetics, esp. malignant hyperthermia, or opioids • Respiratory depression • Cardiovascular instability • Need for induction of general anaesthesia or deep analgosedation • Renal or hepatic impairment • Inability to understand the purpose of the study, perform self-assessments and give verbal informed consent • Degenerative diseases or mental illness that may interfere with pain intensity evaluation • Acute intoxication with drugs or alcohol • Severe head/brain trauma • Life-threatening condition requiring immediate admission to the operating room or intensive care unit • Ongoing use of opioid analgesic agents for chronic pain • Pregnancy or lactation

*NRS* numeric rating scale

### Process

Methoxyflurane (3 ml) was given in one dose via inhaler over a period of no more than 30 min. If methoxyflurane did not achieve a reduction of pain by ≥3 NRS points within 10 min, other analgesics were given intravenously, at the discretion of the emergency physician (piritramide, fentanyl and/or s-ketamine). The study data were recorded by hand or using web-based data entry.

### Statistics

The primary endpoint was effectiveness of methoxyflurane as sole analgesic for transport of patients. Secondary endpoints were user friendliness (EMS personell), time to pain relief, vital parameters, side effects, technical problems, and satisfaction of patients. Due to the design of the trial (observational study of the use of the drug within its approved indications in one group only) analysing the rate of patients without additional analgesia, power analysis was waived. The number of patients was set at 200 plus an allowance for a dropout rate of 5% (total of 210 patients. An interim analysis was planned at 105 patients. However, due to the onset of the severe acute respiratory syndrome coronavirus type 2 (SARS-CoV-2) pandemic, the study essentially came to a halt in March 2020. From that time onwards, concerns about potential transmission of severe SARS-CoV-2 by aerosols from the patients led to avoidance of analgesia by inhalation in favour of intravenous drugs. Inclusion of new patients was finally stopped in November 2020, since the number of patients targeted for the interim analysis had been reached.

The normal distribution of the results was evaluated using a Shapiro-Wilk test. The results of quantitative data are shown as median and interquartile range; frequencies are shown as absolute and percentage values. The patients were divided into the following arbitrary age categories: 18–30, > 30–45, > 45–65, > 65–80, > 80 years. Differences between groups were analysed using Mann-Whitney U tests, Kruskal-Wallis tests, or Jonckheere-Terpstra tests, as appropriate. Frequencies were analysed using Chi-squared or Fisher’s exact tests. A two-sided significance level of *p* < 0.01 was chosen.

## Results

**Fig. 1:**
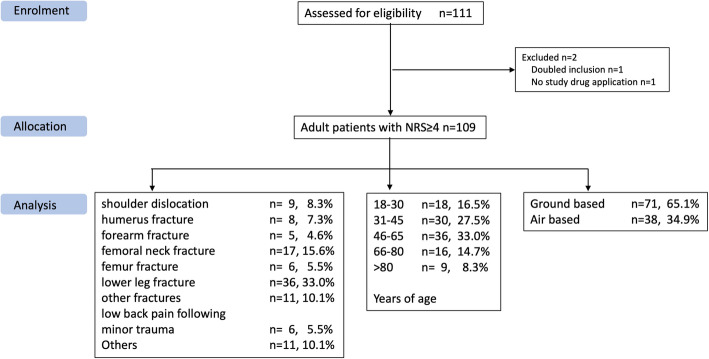
Patient characteristics. n = number of patients; NRS = numeric rating scale

From October 2018 to November 2020, a total of 109 patients were included in the study; two patients were excluded before analysis (one did not need the prepared medication, the other was a double entry). The ratio of men to women was 45.9% (*n* = 50) to 54.1% (*n* = 59); their median age was 51 years (37–64); further population characteristics are listed at Fig. 1 and Table 2.

**Table 2:** A single dose of methoxyflurane: sufficient vs. insufficient analgesia. Patient characteristics and secondary outcome parameters

	Single dose administration of Penthrop®	Insufficient analgesia with Penthrop® or additional analgesics	p
	67	42	
**Population characteristics**			
Sex (female/male)	31/36 (46.3/53.7)	19/23 (45.2/54.8)	0.75
Age	54 (38.0–70.0)	43 (26.8–56.0)	0.01
BMI	24.8 (22.8–27.6)	26.4 (23.4–28.5)	0.31
NRS 0 min	7.0 (6.0–8.0)	8.0 (7.0–9.0)	0.013
HEMS patients	22 (32.8%)	16 (38.1%)	0.58
**Secondary outcome parameters**			
NRS 5 min	5.0 (3.0–6.0)	7.0 (6.0–8.0)	< 0.0001
NRS 10 min	4.0 (3.0–5.0)	6.0 (4.0–7.5)	< 0.0001
NRS 15 min	4.0 (3.0–5.0)	3.5 (2.0–8.0)	0.69
NRS 20 min	4.0 (3.0–5.0)	4.0 (2.0–7.0)	0.66
NRS 30 min	4.0 (2.0–5.0)	4.5 (2.3–8.5)	0.37
Onset of analgesia (min)	3.0 (3.0–4.3)	4.0 (3.0–5.0)	0.22
Duration of treatment (min)	15.0 (10.0–20.0)	10.0 (5.0–18.8)	< 0.0001
User friendliness (EMS personell)	1.0 (1.0–2.0)	1.0 (1.0–3.0)	0.53
User satisfaction (EMS personell)	1.0 (1.0–2.0)	3.0 (2.0–4.0)	< 0.0001
Patient satisfaction with pain therapy	1.0 (1.0–2.0)	3.0 (2.0–5.0)	< 0.0001
Patient satisfaction with prehospital care	1.0 (1.0–1.0)	1.0 (1.0–2.0)	0.081
iv access	15 (22.4%)	42 (100%)	< 0.0001
side effects	32 (47.8%)	26 (61.9%)	0.15
technical problems	10 (14.9%)	6 (14.3%)	0.92

Abbreviations: *BMI* body mass index, *NRS* numeric rating scale, range 0–10; *min* minutes, *i.v.* intravenous, *HEMS* helicopter emergency medical service, user friendliness and satisfaction were measured at a five-point Likert scale with 1 = very good to 5 = bad

The median NRS pain score on arrival of the emergency physician was 8 points (7.0–8.0), falling to 4.0 (3.0–5.0) 15 min after beginning of treatment. The median time to *reduction of pain* (onset of analgesia) was 3.0 (3.0–5.0) minutes; no difference between the sexes was observed in this parameter (women 4.0 min (3.0–5.0 min) versus men (3.0 min (3.0–4.0 min), *p* = 0.14). There was also no significant difference in the time to reduction of pain between the different indications for the emergency callouts.

*Sufficient analgesia* (effectiveness) was achieved using methoxyflurane alone in 67 patients (61.5%); 41 (37.6%) patients needed supplementary analgesia. In one patient therapy was recorded as insufficient but the patient received no additional analgesia. With increasing age of the patients, a significantly better analgesic effect was observed. Due to the supplemental intravenous analgesia in younger patients, this did not manifest as a reduction of NRS scores, but in the highly significant (*p* < 0.0001) decline of the proportion of cases in which methoxyflurane therapy was stopped because of insufficient analgesia. Supplementary analgesia was required in 11/18 (61.1%) of the 18–30 years-old patients, 13/30 (43.3%) of the > 30–45 age group, 13/36 (36.1%) between > 45–65, 3/16 (18.8%) of those > 65–80 and 1/9 (11.1%) of the patients > 80 years old (*p* = 0.044). Patients who received supplemental analgesia were younger (median 43 years, 28.5–56) than patients who did not need supplemental analgesia (53.5 years, 38–70) (*p* = 0.01). There were no significant differences by sex (18/23, 43.9/56.1% versus 32/36, 47.1/52.9%, *p* = 0.75), BMI (26.4 (23.4–28.5) versus 24.8 (22.8–27.6) (*p* = 0.31), by the indications for the emergency callout (as listed in Fig. 1, *p* = 0.13) or the time to onset of the drug effect (4.0, 3.0–5.0 vs. 3.0 3.0–4.3, *p* = 0.22). Patients who did not require supplemental analgesia tended to have lower initial pain scores, although the difference, of only one NRS point, was clinically marginal: 7.0 (6.0–8.0) vs. 8.0 (7.0–8.5, *p* = 0.013).

In all cases, the patients’ *vital parameters* were stable throughout prehospital care, and, if anything, slightly elevated due to stress and pain: the median blood pressure was 140/80 mmHg (125–150/80–90 mmHg), the median pulse rate was 85 bpm (78–94), peripheral oxygen saturation was 97% (96–99%). The median score of 15 (15–15) on the Glasgow Coma Scale was also normal. On application of Penthrop®, with the resulting analgesia, blood pressure and pulse rate dropped slightly, i.e. the cardiovascular parameters normalized, without any clinically relevant effect on oxygen saturation and consciousness.

The *patients’ satisfaction* with the pain treatment and care by the emergency team was very good (Table 2).

When the patients were proactively asked about *side effects* of the therapy, 53.2% (*n* = 58) of them reported that they had experienced them, but mostly described them as mild (Table 3). With increasing age, the incidence, but not the severity, of side effects increased in tendency (*p* = 0.099). Only in one case (0.9%), therapy was stopped because of nausea and the unpleasant odour. Five patients (8.6%) in total reported an unpleasant taste or smell. No adverse event occurred during the study.

**Table 3:** Adverse effects associated with administration of methoxyflurane (Penthrop®)

Side effects	n	%
**Dizziness**	23	21.1%
**Confusion**	10	9.2%
**Feeling drunk**	9	8.3%
**Combined side effects**	9	8.3%
**Disgusting taste/smell**	5	4.6%
**Sedation**	5	4.6%
**Nausea**	4	3.6%
**Malignant hyperthermia**	0	0.0%
**Vomiting**	0	0.0%

*n* = 58 (53.2%), multiple entries possible

The emergency physicians rated the *user friendliness* (EMS personell) of the inhaler as very good (median 1.0 (1.0–2.0)). The overall user satisfaction (EMS personell) was good (median 2.0 (1.0–3.0). Nevertheless, technical difficulties were reported in 14.7% (*n* = 16) of cases: although users followed the device instructions, in some cases liquid leaked from the inhaler. In these cases, the emergency personnel reported unpleasant odours, especially in confined spaces (helicopter or ambulance cabins, *n* = 22, 20.2%). This affected the scoring: in these cases, the median user satisfaction score was only 3.0 (2.0–4.0).

## Discussion

In this prospective observational study, the inhaled analgesic methoxyflurane (Penthrop®) was found to be effective in 61.5% (67 of 109) patients with with moderate to severe pain due to fractures, dislocations and low back pain due to minor trauma. Side effects were frequent (53%) but mild with just one truncation of therapy (0.9%) due to side effects. The original plan to include 200 patients in the study had to be abandoned because of the SARS-CoV-2 pandemic. The study was stopped because of the risk of aerosol transmission due to the inhalation-based delivery method.

However, the number of cases collected corresponds to the planned number for an interim analysis, and the amount of data collected is sufficient to draw some conclusions on the main questions. Almost two-thirds of patients who initially had very severe pain (median NRS 8) experienced a significant reduction in pain of 4 NRS points within 15 min of methoxyflurane. This corresponds to or is even better than reports to date from European emergency departments [28–30]. The onset of the analgesic effect was rapid, the emergency physicians rated the ease of use of the product as good and patients rated the analgesia as good. The effectiveness of the drug was noticeably, and in clinical terms significantly, better in older patients (no supplemental analgesia required for > 87% of the patients over 65 years old). This resulted in higher satisfaction of older patients despite a higher incidence of side effects. On the other hand, younger patients experienced insufficient analgesia more often, independent of sex and indication: half of the patients under 45 needed additional analgesia. This can be explained by the fact that the minimal alveolar concentration (MAC) of all inhaled anaesthetics declines with increasing age. It might be worthwhile for manufacturers to consider a way to provide variable doses for different age groups. This might improve the acceptance and applicability of the drug in younger patients.

**Fig. 2:**
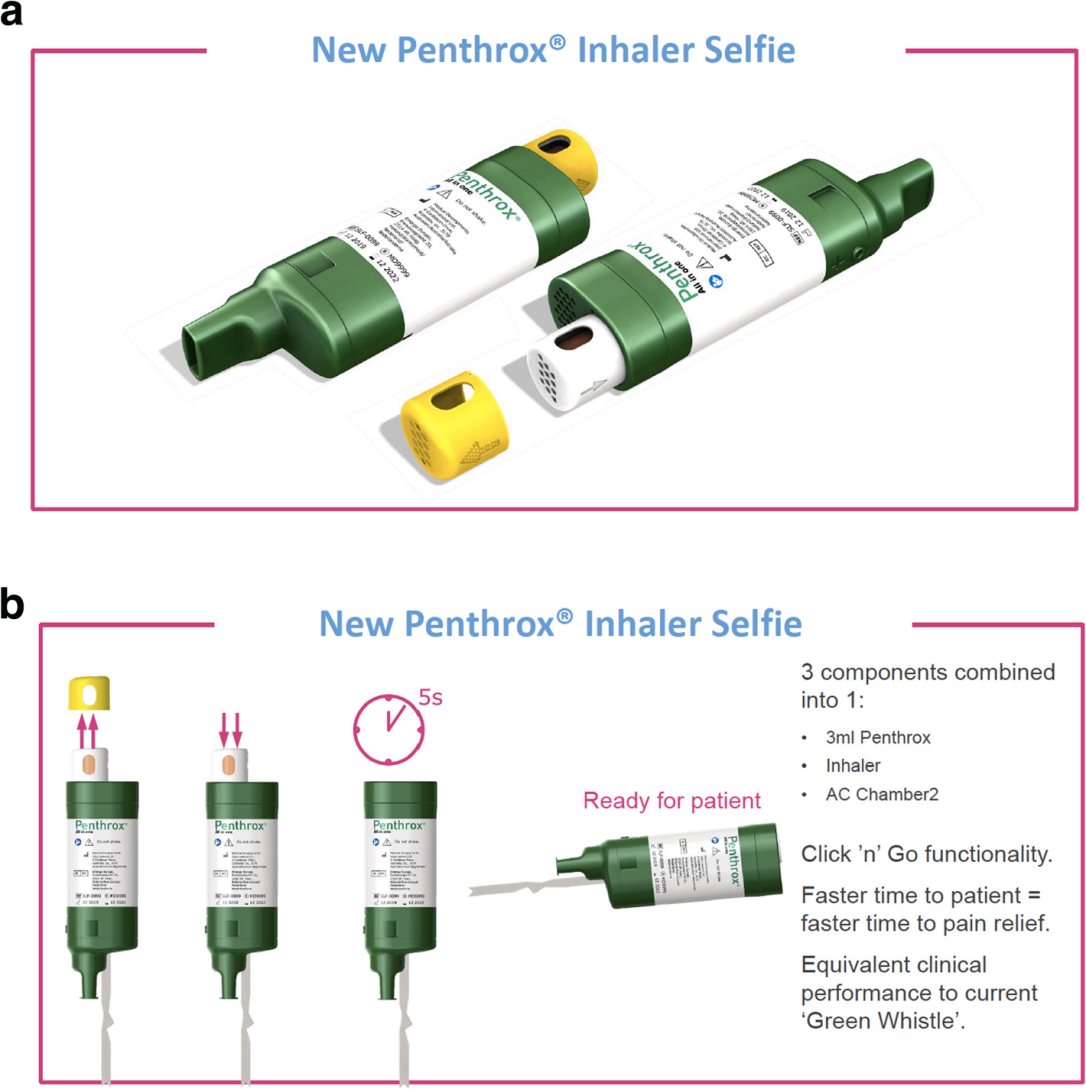
**a** New Penthrox® inhaler “selfie”. The new device will already include the active ingredient. **2b** Penthrox® inhaler “selfie” – how to use. Pressing the button releases the active ingredient onto the gauze and the device is ready for administration after 5 s

Methoxyflurane is certainly easier to handle than nitrous oxide [11, 20, 33]. However, the patients, especially if they are older, have to be carefully instructed how to use the inhaler; this is not necessarily easy to do in the acute situation with a patient in pain. The effectiveness depends on whether the patient has understood the instructions correctly and is able to implement them. The possibility of increasing the concentration by closing the ‘dilutor hole’ should only be mentioned after the patient has had a little time to get used to the drug, because if they inhale it at full concentration right away, they are likely to experience the taste as unpleasant (8.6%) and may reject the treatment. After five or six breaths this phenomenon usually disappears and the analgesia already becomes noticeable. Preparation of the inhaler in the current version was slightly vulnerable to errors: in one use in every seven, the filling step led to liquid leaking out of the mouthpiece onto the floor. This could impair the effectiveness of the drug or shorten the period one dose will last for. A new design of the device intended to prevent this problem is in development (Fig. 2a and b, „Penthrox® Inhaler Selfie“) and should be available in 2022 at the latest (verbal communication). The new inhaler will be ready-loaded with the drug vial and will no longer need to be filled manually. This would also reduce the unpleasant odour complained of by one in five users, which was also commented on as a particular nuisance by the helicopter crews (29% vs. 15%).

The side effects of Penthrop® are known; the substance has been proven to be safe and effective over many studies [11, 25]. In agreement with previous reports, the most common side effects reported by patients, on being asked, were feelings of mild dizziness, confusion and/or inebriation (Table 3). Interestingly, the patients tended to experience these side effects as not unwelcome. Only in one case (0.9%) was it necessary to stop the treatment because of a side effect (nausea) and the unpleasant taste. No relevant effects on vital functions were observed. And although methoxyflurane is less effective than intravenous or intranasal opioids [24], the ease of administration, logistics and documentation, combined with the legal restrictions on administration of opioids by non-physician personnel, speak for the licensing of this drug for pre-hospital use by paramedical personnel in Europe. Often the need for rapid analgesia is needed most for patient repositioning manoeuvres: once the positioning is complete, further analgesia is often not crucial. Effective and safe analgesia by paramedics could reduce the number of callouts of emergency physicians and improve their availability for other, vitally endangered patients. In fact, in our study, almost half the patients served by ground ambulances were handed over to the paramedical emergency team without the need to place an intravenous line or give further analgesic medication. This explains the relatively short median attendance time of 34 min (15–85). Multiplied by the total number of callouts for the indication ‘moderate to severe pain’ this would indicate considerable potential for cases that could be managed by the non-physician emergency teams [2]. Penthrop® is also an interesting option for alpine mountain rescue. Whenever the time to arrival of an emergency physician is going to be longer, and in adverse conditions, such as winter weather, an effective form of analgesia without the need for an intravenous line would be a valuable addition to the paramedical emergency repertoire. The simple and quick availability can also be an advantage to emergency physicians: the self-titration by the patient was especially useful for rescues in difficult terrain, in alpine settings, and during transport in a rescue bag.

### Limitations of the study

The main limitation of this trial is the relatively low number of patients. Unfortunately, the premature end of the study due to the pandemic prevented the collection of further observations, e.g. in alpine air rescue operations. However, since the results obtained up to this point answered our primary questions sufficiently, the study was closed. Further investigations in this field will follow. Due to the absence of information about potential viral spreading via inhaler technique, we can neither recommend nor advise against this device during the current or future pandemics. Methoxyflurane was not tested against a placebo, since its analgesic effects are already sufficiently described [34]. Our study focused on a first prehospital application under the specific conditions of a Central European emergency service with emergency physicians. For ethical reasons it was not possible to avoid mixing of results for pain reduction with and without supplemental analgesia (usually opioids with or without S-ketamine), since the patients have a right to adequate pain relief.

## Conclusions

In prehospital use, inhaled methoxyflurane as sole analgesic is effective for transport of trauma patients (62%) with moderate to severe pain. During the SARS-CoV-19 pandemic or similar situations, the use of an inhaler can be seen as precarious. Especially older patients benefit from inhaled methoxyflurane. Side effects are mild and vital parameters are unaffected. Thus, inhaled methoxyflurane could be a valuable option for non-physician rescue services in Central Europe.

## Data Availability

All data that support the findings of this study are available from the corresponding author upon reasonable request.

## Abbreviations

BMI: Body mass index
CRF: Case report form
EMA: European medicines agency
EMS: Emergency medicine service
GCS: Glasgow coma scale
MAC: Minimal alveolar concentration
NRS: Numeric rating scale
SARS-CoV-2: Severe acute respiratory syndrome coronavirus type 2
